# Design, Synthesis, Antinociceptive and Anti-Inflammatory Activities of Novel Piroxicam Analogues

**DOI:** 10.3390/molecules171214126

**Published:** 2012-11-28

**Authors:** Amanda Silva de Miranda, Walfrido Bispo Júnior, Yolanda Karla Cupertino da Silva, Magna Suzana Alexandre-Moreira, Rosane de Paula Castro, José Ricardo Sabino, Luciano Morais Lião, Lídia Moreira Lima, Eliezer J. Barreiro

**Affiliations:** 1 LASSBio® – Laboratório de Avaliação e Síntese de Substâncias Bioativas, Faculdade de Farmácia, Universidade Federal do Rio de Janeiro, P.O. Box 68024, 21944-971, Rio de Janeiro, RJ, Brazil; 2 Pós-graduação em Química, Instituto de Química, Universidade Federal do Rio de Janeiro, 21941-909, Rio de Janeiro, RJ, Brazil; 3 LaFI – Laboratório de Farmacologia e Imunidade, Instituto de Ciências Biológicas e da Saúde, Universidade Federal de Alagoas, 57072-970, Maceió, AL, Brazil; 4 RENORBIO, Rede Nordeste de Biotecnologia, Universidade Federal de Alagoas, 57072-970, Maceió, AL, Brazil; 5 Instituto de Física, Universidade Federal de Goiás, 74001-970, Goiânia, GO, Brazil; 6 Instituto de Química, Universidade Federal de Goiás, 74001-970, Goiânia, GO, Brazil

**Keywords:** piroxicam, inflammation, pain, acylhydrazone, NSAID, COX

## Abstract

In this paper we report the design, synthesis, antinociceptive and anti-inflammatory activities of a series of benzothiazine *N*-acylhydrazones **14a**–**h**, planned by structural modification of piroxicam (**1**), a non steroidal anti-inflammatory drug. Among the synthesized analogues, compounds **14f** (LASSBio-1637) and **14g** (LASSBio-1639) were identified as novel antinociceptive and anti-inflammatory prototypes, active by oral administration, acting by a mechanism of action that seems to be different from that of piroxicam, since they were inactive as an inhibitor of cyclooxygenase (COX-1 and COX-2) at concentrations of 10 μM.

## 1. Introduction

Inflammation is an adaptive response triggered by trauma, tissue injury or infection, characterized by symptoms such as pain, redness, heat and swelling. Acute inflammation is mediated mainly by the release of prostaglandins and other pro-inflammatory mediators, like leukotrienes, bradykinin and histamine, which promote local vascular alterations and leukocyte recruitment and activation. This process is normally self limited and resolution occurs with elimination of the noxious stimuli, removal of inflammatory cells and tissue repair. However, under certain conditions the inflammatory process persists, leading to a chronic inflammatory process, which additionally involves interleukins, tumor necrosis α, interferons, among other mediators, and underlines a variety of chronic diseases, such as cancer, atherosclerosis and reumathoid arthiritis [1,2,3]. Currently drug management of inflammation includes non steroidal anti-inflammatory drugs (NSAIDs), glucocorticoids, slow-acting disease-modifying anti-rheumatic drugs (DMARDs), immunosuppressants and biologics that specifically target inflammatory cytokines [3]. 

**Figure 1:**
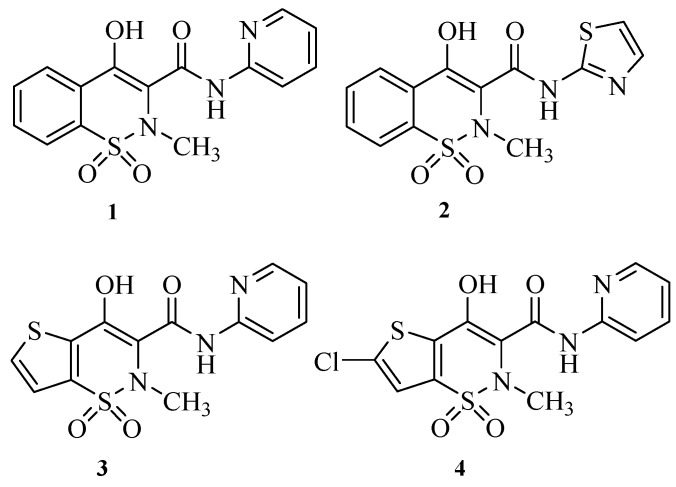
Oxicam class drugs.

Piroxicam (4-hydroxy-2-methyl-2*H*-1,2-benzothiazine-1-(*N*-(2-pyridinyl)carboxamide)-1,1-dioxide), (**1**) [4] is a NSAID discovered in 1972 and introduced in the market by Pfizer in 1982, being the first drug of the “oxicam” class [5], to which meloxicam (**2**) [6], tenoxicam (**3**) [7] and lornoxicam (**4**) [8] (Figure 1) also belong. Piroxicam is a long half-life drug (45 h in humans) [9] which also possess analgesic and antipyretic properties and has been used in the management of chronic inflammatory diseases [10], such as rheumatoid arthritis and osteoarthritis, for almost 30 years. Like other NSAIDs, the therapeutic effects of piroxicam have been attributed to its ability to inhibit cyclooxygenase (COX), [11,12] a key enzyme in the biosynthesis of pro-inflammatory prostanoids, such as prostaglandins. 

**Figure 2:**
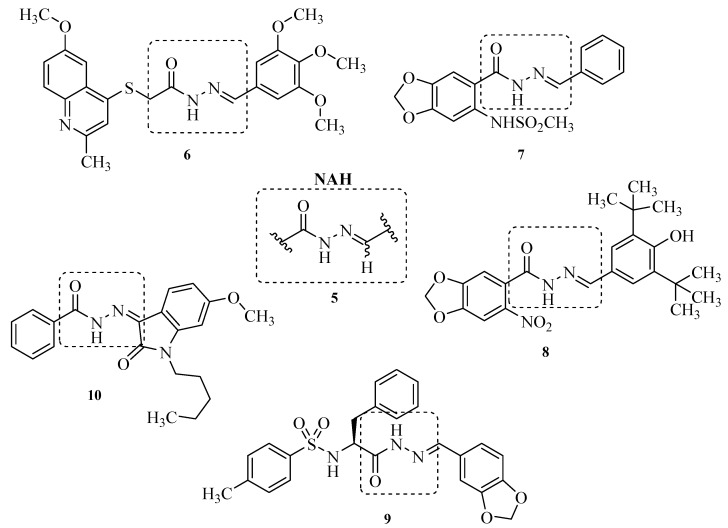
*N*-acylhydrazones derivatives with different activities.

The *N*-acylhydrazone (NAH; **5**) framework has recently been recognized as a privileged structure [13,14,15] and several NAH derivatives (Figure 2) have been reported with different pharmacological activities, such as PDE inhibitors **6** [16] COX inhibitors **7** [17], TRPV1 antagonists **8** [18], HIV-1 capsid protein assembly inhibitors **9** [19], and CB2 inverse agonists **10** [20], among others. Moreover, many *N*-acylhydrazones were described by our research group as potent anti-inflammatory and antinociceptive compounds [21], which have been attributed to the NAH framework’s ability to mimic amide group and the bis-allylic moiety of unsaturated fatty acids, such as arachidonic acid [22,23].

**Figure 3:**
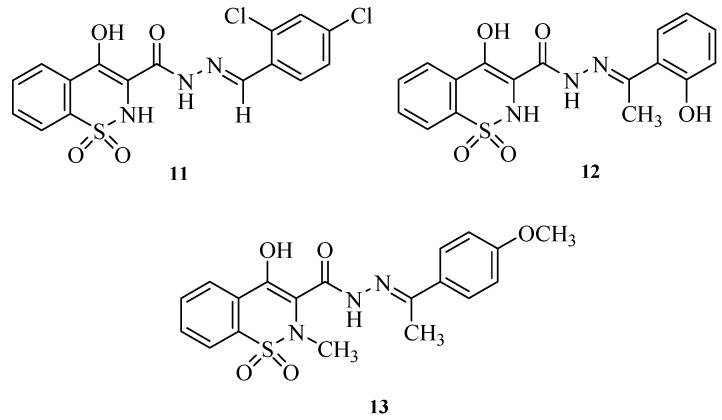
Antioxidant and antimicrobial *N*-acylhydrazones bearing a 4-hydroxy-1,2-benzothiazine-1,1-dioxide scaffold.

Very recently, some antioxidant and antimicrobial *N*-acylhydrazones bearing a 4-hydroxy-1,2-benzothiazine-1,1-dioxide scaffold were reported [24,25,26] (Figure 3). Nevertheless, to the best of our knowledge, the antinociceptive and anti-inflammatory potential of this molecular pattern has not been investigated. Besides, some structural features that seems to be related to the oxicams’ anti-inflammatory activity, such as heteroaromatic amide subunit [4,27], have not been fully explored in these previous works.

**Figure 4:**
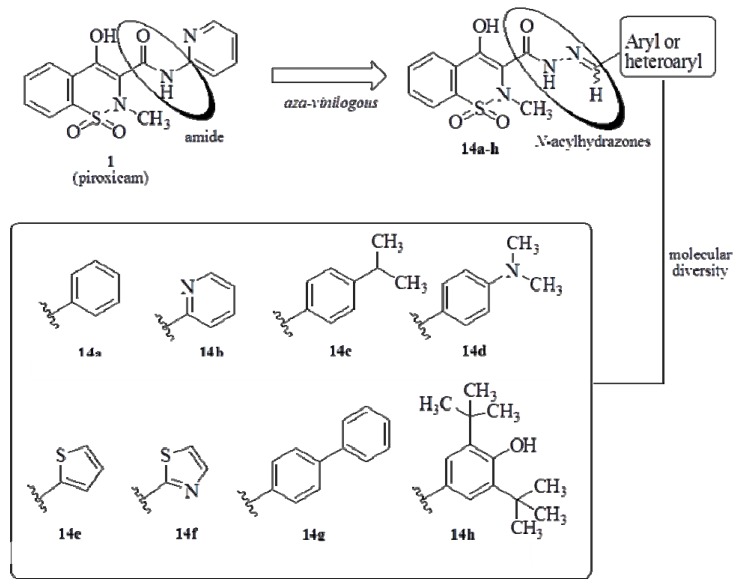
Design of a novel series of analgesic and anti-inflammatory benzothiazine *N*-acylhydrazones.

In this paper, we report the design, synthesis and pharmacological evaluation of a novel series of antinociceptive and anti-inflammatory *N*-acylhydrazones derivatives (all compounds are original, with the exception of **14a**, which was first published by Zia-ur-Rehman *et al*. [24]). Target compounds **14a**–**h** were designed (Figure 4) to include a 4-hydroxy-1,2-benzothiazine-1,1-dioxide scaffold, a pharmacophore present in oxicam drugs, such as pioxicam, and the NAH framework, a privileged structure which is also encountered in many anti-inflammatory compounds [21]. The nature of the aryl and heteroaryl groups attached to the imine subunit was varied based on ring isosteric replacement to give **14a**–**f**. In addition, we have synthesized compound **14g**, which bears a biphenyl group, a pharmacophore found in some recently reported mPGES-1 inhibitors [28] and also considered aprivileged structure [13,14]. In order to confer antioxidant and radical scavenging properties we designed compound **14h**, featuring a 3,5-di-*tert*-butyl-4-hydroxyphenyl group, a subunit found in many dual cyclooxygenase/5-lipoxygenase (COX/5-LOX) inhibitors [29]. 

## 2. Results and Discussion

### 2.1. Chemistry

**Scheme 1:**
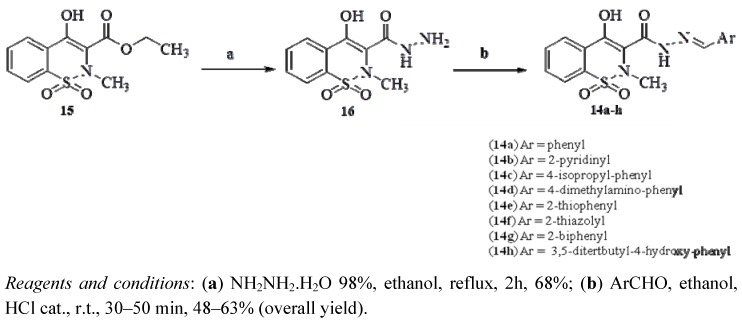
Synthesis of compounds **14a**–**h**.

*N*-acylhydrazones **14a**–**h** were synthesized from commercial available ethyl 4-hydroxy-2*H*-1,2-benzothiazine-3-carboxylate 1,1-dioxide [30] (**15**) in two steps, as depicted in Scheme 1. The key intermediate **16** was obtained in 68% yield by treating an ethanolic solution of **15** with 98% hydrazine monohydrate for 2 h under reflux [31]. Finally, condensation of **15** with appropriate aromatic and heteroaromatic aldehydes at room temperature, under acid catalysis [31], provided the target compounds **14a**–**h** in 48–63% overall yield.

The novel *N*-acylhydrazone derivatives **14a**–**h** were fully characterized by spectroscopic methods, such as IR, ^1^H and ^13^C-NMR, their purities were established by HPLC and determined by elemental analysis (CHN). Their molecular formulae were confirmed by mass spectroscopy (ESI-MS). All compounds were obtained as a single tautomer, which was found to be the enolic form, as indicated by signals at δ 8.52–8.85 (N=C-*H*), 11.65–12.23 (CON*H*) and δ 13.93–14.42 ppm (O-*H*) in the ^1^H-NMR spectra in DMSO-*d*_6_ and by signals at δ 110.56–111.23 (*C*=C-OH), 144.70–152.42 (*C*=N), 157.56–163.76 (*C*-OH) and 164.51–165.98 ppm (*C*=O) in the ^13^C-NMR spectra recorded in the same solvent. The predominance of enolic tautomer is in agreement with recently published data [24,25,26]. Besides, the presence of only one peak in HPLC chromatogram and a single imine-hydrogen signal in the ^1^H-NMR spectra suggest that condensation reaction was diastereoselective. Carefully comparison of amide and imine hydrogens chemical shift of compounds **14a**–**h** with previous published NMR data for *N*-acylhydrazones whose stereochemistry had been established by crystallography [17,32,33] suggested the presence of the *E*-diastereoisomer, which was confirmed by performing a NOE experiment on compound **14b**. Thus, on irradiating at the frequency of the amide hydrogen (CON*H*) in **14b**, a positive NOE was observed for the signal at δ 8.71 ppm, assigned to imine hydrogen (C=N-*H*), and a positive NOE was also observed for the signal at δ 2.89 ppm (C*H*_3_) on irradiating imine hydrogen (N=C-*H*), thus indicating the *E*-configuration for C=N double bond. The predominance of the *E* diastereoisomer of *N*-acylhydrazone derivatives is consistent with previous works [17,31,32,33,34,35,36,37] and has been ascribed to the greater thermodynamic stability of the *E* in respect to the *Z* diastereoisomer [36,38].

**Figure 5:**
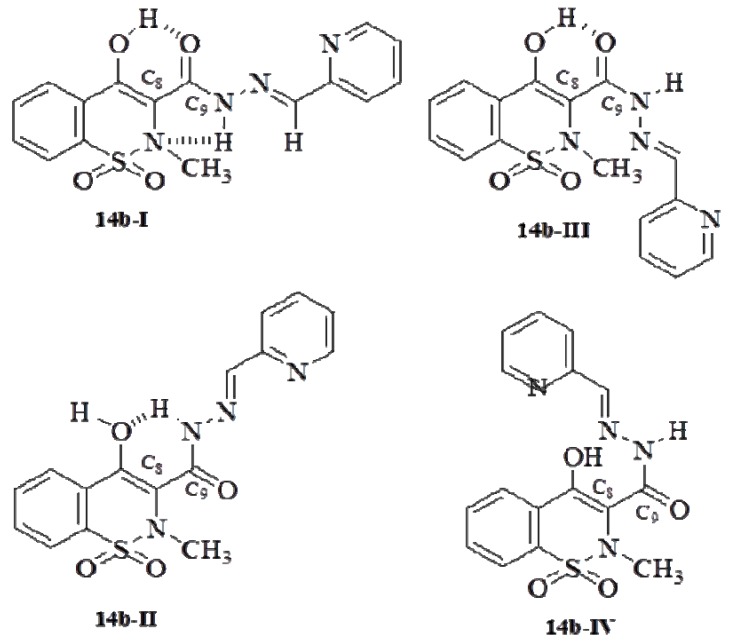
Possibles conformations of compound **14b** (LASSBio-1617).

In addition, an intense NOE observed for amide hydrogen on irradiating the methyl hydrogens suggests that a close flat conformation, featuring an *S*-cis conformation for C8-C9 bond and amide hydrogen antiperiplanar to carbonyl oxygen (**14b-I**), predominates over an open or folded conformation, featuring an *S*-trans C8-C9 bond (**14b-II**) or an amide hydrogen antiperiplanar to carbonyl oxygen (**14b-III**) or both (**14b-IV**) (Figure 5). The predominance of conformer **14b-I** is likely to be due to additional stability provided by hydrogen bonds between the enolic and carboxyl groups and amide hydrogen and benzothiazine nitrogen lone electron pair (Figure 5). These results are in agreement with previous works [24,32] and are likely to reflect also the stereochemical and conformational features of the other compounds of the series. 

**Figure 6:**
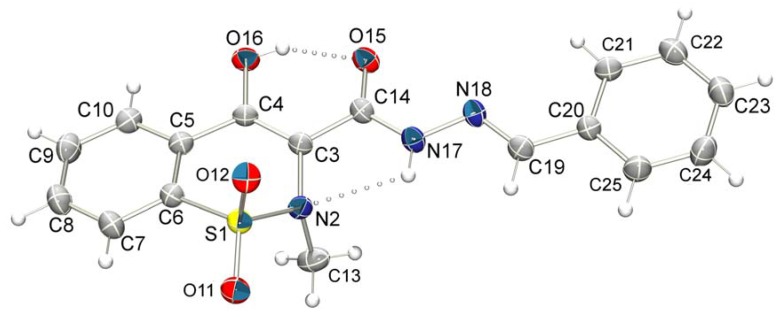
ORTEP view of compound **14a** (LASSBio-1606) with atom displacement ellipsoids drawn at 30% probability.

In order to confirm the results mentioned above and to determine unambiguously the relative configuration of compounds **14a**–**h**, the single crystal of compound **14a** (LASSBio-1606**)** was obtained for X-ray diffraction. The ORTEP [39] view of compound **14a** is shown in Figure 6. Crystallographic analysis confirmed the *E* configuration and revealed a flat conformation, described by the least squares plane through the atoms N2/C3/C4/O16/O15/C14/N17 with mean deviation from the plane of 0.07 Å, featuring the intra-molecular hydrogen bonds involving N17—H…N2 and O16—H…O15, with donor-acceptor distances 2.723(3) Å and 2.541(3) Å, respectively, corroborating the conclusions suggested by NOE experiment performed with **14b**.

### 2.2. Pharmacology

Compounds **14a**–**h** were evaluated *in vivo* for their antinociceptive and anti-inflammatory activities at the screening dose of 100 µmol/kg. All compounds were administrated orally and piroxicam was used as standard drug, except in the hot plate test, for which morphine was used instead. The antinociceptive activity of compounds **14a**–**h** was initially evaluated employing the acetic acid-induced abdominal writhing model in mice [40]. As shown in Table 1, all derivatives produced marked inhibition of the acetic acid-induced writhing response. Compound **14e** (LASSBio-1604) (90.4%), which bears a 2-thiophenyl group, the biphenyl analogue **14g** (LASSBio-1638) (84.0%) and **14h**, featuring a 3,5-di-*tert*-butyl-4-hydroxyphenyl group (LASSBio-1639) (82.4%), were found to be the most active compounds and showed an antinociceptive effect similar to that of piroxicam (95.4%) at 100 µmol/kg. 

**Table 1:** Effect of compounds **14a**–**h** and piroxicam (100 µmol/kg, p. o.) on acetic acid-induced writhing test in mice.

Compound	Writhing Number ^a^	% of Inhibition
Control	39.4 ± 4.9	-
Piroxicam	1.8 ± 0.6 **	95.4%
**14a** (LASSBio-1606)	9.4 ± 1.6 *	76.1%
**14b** (LASSBio-1617)	16.3 ± 2.5 *	58.6%
**14c** (LASSBio-1605)	9.8 ± 5.1 *	75.1%
**14d** (LASSBio-1607)	16.6 ± 2.9 **	57.9%
**14e** (LASSBio-1604)	3.8 ± 1.7 **	90.4%
**14f** (LASSBio-1637)	14.7 ± 2.6 **	62.7%
**14g** (LASSBio-1638)	6.3 ± 1.5 **	84.0%
**14h** (LASSBio-1639)	6.8 ± 2.3 **	82.7%

^a^ The readings represent the mean ± S.E.M. The asterisks denote the significance levels in comparison with control groups (* *p* < 0.05, ** *p* < 0.01).

A dose-response curve was obtained for derivatives **14e**, **14g** and **14h** (Table 2). Considering that compounds **14b** (LASSBio-1617) and **14f** (LASSBio-1637) present the major structural similarity to the standard drug piroxicam, they were also selected for the dose-response study. Compounds **14b **and **14e**–**h** produced dose-related inhibition of acetic acid-induced abdominal constrictions in mice and their ID_50_ and maximum efficacy are summarized in Table 2.

**Table 2:** Maximum efficacy and ID_50_ values of compound **14b**, **14e**–**h** and piroxicam on acetic acid-induced writhing test in mice.

Compound	ID_50_ (µmol/kg)	Maximum efficacy
piroxicam	0.40 (0.0013–115.6)	95.4%
14b (LASSBio-1617)	115.6 (0.17–808.30)	86.00%
14e (LASSBio-1604)	28.57 (6.29–49.61)	90.30%
14f (LASSBio-1637)	17.19 (0.189–156.1)	84.77%
14g (LASSBio-1638)	14.07 (1.06–187.3)	80.20%
14h (LASSBio-1639)	2.87 (0.06–130.5)	88.83%

None of the NAH derivatives were found to be more potent than piroxicam (ID_50_ = 0.40 µmol/kg) in the acetic acid-induced writhing test, since the most potent antinociceptive in this series was **14h** (LASSBio-1639, ID_50_ of 2.87 µmol/kg). Nevertheless, all compounds showed maximum efficacy in the 80–90% range, like the standard drug piroxicam (95.4%). In order to better understand the antinociceptive effect showed by NAH derivatives, the formalin-induced pain test in mice [41] was carried out (Table 3). Formalin is known to produce a biphasic pain behavior. The first transient phase, or neurogenic phase, is ascribed to the direct effect of formalin on sensory C fibers, and the second prolonged phase, also called inflammatory phase, is associated to the development of an inflammatory response and the release of analgesic mediators [41,42,43]. With the exception of compound **14b** (58%), none of NAH derivatives were shown to be active in the neurogenic phase of the formalin test (data not shown). On the other hand, at 100 µmol/kg, compounds **14b**, **14e**–**h** were able to inhibit the pain response in the inflammatory phase, with compounds **14f** (LASSBio-1637; 60.1%) and **14g** (LASSBio-1638; 54.2%), which presented similar antinociceptive effect than piroxicam (53.9%) standing out. Their lack of effect in the neurogenic phase and expressive inhibition of the inflammatory phase of the formalin test, comparable with the response of piroxicam and NSAIDs in general, suggest that these new NAH derivatives are likely to produce their antinociceptive effect by acting on the inflammatory process.

**Table 3:** Effect of piroxicam NAH analogues (**14a**–**h**) and piroxicam (100 µmol/kg, p.o.) on formalin-induced pain test in mice.

Compound	Phase 1	Phase 2	% of inhibition Phase 2
Control	50.9 ± 5.8	194.0 ± 10.5	-
Piroxicam	50.0 ± 5.8	89.3 ± 22.8 **	53.9%
**14a** (LASSBio-1606)	49.6 ± 11.2	172.5 ± 25.9	11.1%
**14b** (LASSBio-1617)	21.3 ± 5.2 *	119.6 ± 23.9 *	38.4%
**14c** (LASSBio-1605)	36.1 ± 6.2	176.3 ± 21.9	9.1%
**14d** (LASSBio-1607)	38.8 ± 8.8	161.4 ± 27.9	16.8%
**14e** (LASSBio-1604)	49.6 ± 10.7	131.0 ± 13.1 **	32.5%
**14f** (LASSBio-1637)	50.3 ± 10.6	77.5 ± 16.7 *	60.1%
**14g** (LASSBio-1638)	56.6 ± 10.5	88.8 ± 18.5 **	54.2%
**14h** (LASSBio-1639)	54.8 ± 5.7	140.7 ± 13.9 *	27.5%

The readings represent the mean ± S.E.M. The asterisks denote the significance levels in comparison with control groups (* *p* < 0.05, ** *p* < 0.01).

In order to determine any eventual central antinociceptive activity for compounds **14a**–**h**, the hot plate test [31] was carried out in mice. This test is used to evaluate antinociceptive activity mediated by a central mechanism and it is selective for opioid drugs, although sensitive to some sedatives, hypnotics and muscle relaxants [44,45,46,47]. None of NAH derivatives were found to be active in this test (data not shown), even **14b** (LASSBio-1617), which produced a weak inhibition of pain response in the neurogenic phase of formaline test. Piroxicam was also inactive, as expected. These results reinforce that compounds **14a**–**h** do not have any central effect and that their antinociceptive effect is mediated peripherically, thus confirming the results found in the formalin-induced pain test. To confirm the anti-inflammatory profile of NAH derivatives, as suggested by the results obtained in the formalin test, zymosan A [48,49] and carrageenan-induced peritonitis test in mice [50] were performed. As shown in Table 4, all NAH derivatives significantly inhibited cell recruitment in the zymosan-induced peritonitis test and, with exception of **14e** (37.1%) and **14a** (43.7%), all compounds were found to be similar or more active than piroxicam (57.4%) at the dose of 100 µmol/kg, highlighting compounds **14g** (LASSBio-1638) and **14h** (LASSBio-1639), which inhibited 81.3% and 82.6% of cell migration, respectively.

**Table 4:** Effect of piroxicam NAH analogues and piroxicam (100 µmol/kg, p.o.) on zymosan-induced peritonitis in mice.

Compound	Cell Number X 10^6^/mL	% of inhibition
Control	38.0 ± 1.0	-
Piroxicam	16.2 ± 1.1 **	57.4%
**14a** (LASSBio-1606)	21.4 ± 2.4 *	43.7%
**14b** (LASSBio-1617)	9.9 ± 0.7 **	73.9%
**14c** (LASSBio-1605)	16.5 ± 3.7 **	56.6%
**14d** (LASSBio-1607)	10.6 ± 0.4 **	72.1%
**14e** (LASSBio-1604)	23.9 ± 1.7 **	37.1%
**14f** (LASSBio-1637)	8.1 ± 1.8 **	78.7%
**14g** (LASSBio-1638)	7.1 ± 0.6 **	81.3%
**14h** (LASSBio-1639)	6.6 ± 1.3 **	82.6%

The readings represent the mean ± S.E.M. The asterisks denote the significance levels in comparison with control groups (* *p* < 0.05, ** *p* < 0.01).

In the carrageenan-induced peritonitis test (Table 5) all NAH derivatives were able to inhibit cell migration similarly or more effectively than piroxicam (25.8%), including **14c** (LASSBio-1605; 62.2%), **14a** (LASSBio-1606; 57.4%) and **14d** (LASSBio-1607; 42.3%), which were not active in the second phase of formalin-induced pain test. 

**Table 5:** Effect of piroxicam NAH analogues and piroxicam (100 μmol/kg, p.o.; mean ± S.E.M.) on carrageenan-induced peritonitis in mice.

Compound	Cell Number X 10^6^/mL	% of inhibition
Control	20.9 ± 1.6 **	-
Piroxicam	15.5 ± 0.3 **	25.8%
**14a** (LASSBio-1606)	8.9 ± 0.5 **	57.4%
**14b** (LASSBio-1617)	16.2 ± 0.7 **	22.5%
**14c** (LASSBio-1605)	7.9 ± 0.8 **	62.2%
**14d** (LASSBio-1607)	12.0 ± 1.5 **	42.6%
**14e** (LASSBio-1604)	5.4 ± 0.6 **	74.2%
**14f** (LASSBio-1637)	5.6 ± 0.5 **	73.2%
**14g** (LASSBio-1638)	4.7 ± 0.7 **	77.5%
**14h** (LASSBio-1639)	3.8 ± 0.6 **	81.8%

The asterisks denote the significance levels in comparison with control groups (* *p* < 0.05, ** *p* < 0.01).

LASSBio-1639 (**14h**) and LASSBio-1638 (**14g**) were the most active compounds, inhibiting 81.8% and 77.5% of cell migration, respectively, followed by LASSBio-1604 (**14e**), which, despite being the least active compound in zymosan-induced peritonitis test, showed inhibition of 74.2% in carrageenan-induced peritonitis test. 

In order to investigate if the anti-inflammatory effect of NAH derivatives were COX inhibition- dependent, like piroxicam, compounds **14b** and **14f** were selected for *in vitro* assay for their ability of inhibiting COX-1 and COX-2 isoforms [51]. Despite their antinociceptive and anti-inflammatory activities, *in vivo*, none of the compounds were able to inhibit COX at concentration of 10 μM, suggesting that they may act in a different way than NSAIDs (data not shown) or would be significantly less potent than the prototype piroxicam.

## 3. Experimental

### 3.1. General

Reagents and solvents were obtained from commercial sources (Sigma-Aldrich, Alfa Aesar) and used as received. Reaction progress was monitored by thin-layer chromatography (TLC) on commercial silica gel plates (KieselGel 60 F245 on aluminum sheets, Merck) and visualized by UV-light (254 and 365 nm). Column chromatography purifications were carried out using silica gel Merck 230–400 Mesh. Melting points were determined on a Quimis Q240.23 apparatus and are uncorrected. ^1^H- and ^13^C- nuclear magnetic resonance spectra were recorded in DMSO-*d*_6_ solutions on a Bruker Avance 200 MHz or on a Varian 400 MHz instrument. Chemical shift (δ) are given in parts per million (ppm) down field from tetramethylsilane (TMS) and couple constants (*J*) are given in Hertz (Hz); splitting patterns are reported as follows: s, singlet; d, doublet; dd, doublet of doublets; ddd, doublet of doublets of doublets; t, triplet; q, quartet; m, multiplet; br, broad. Electrospray ionization mass spectra (ESI-MS) were acquired using a Micromass quadrupole-time-of-flight (QTOF) spectrometer operating in a positive mode. Infrared (IR) spectra were performed in bromide potassium (KBr) disks on Nicoleta Magna IR 760 spectrometer. HPLC analysis were performed on a Shimadzu 20A using a C18 column (4,6 mm × 250 mm, 5 μM) eluted with MeCN/H_2_O (90:10;70:30 or 45:55) over 15 or 20 min at flow rate of 1 mL·min^−1^, with UV detection at 254 nm. 

*4-Hydroxy-2-methyl-2H-1,2-benzothiazine-3-carbohydrazide 1,1-dioxide* (**16**). A mixture of compound **15** (2.000 g, 7.06 mmol), and hydrazine hydrate 80% (6.8 mL, 137.4 mmol) in ethanol (40,0 mL) was stirred under reflux for 2h, when completion of reaction was indicated by TLC. The mixture was partially concentrated under vacuum, followed by addition of water and HCl 37% until precipitation. The solid was filtered and washed with water and cold ethanol to furnish compound **22** (1.256 g, 68%), which had the following properites: Mp 198–199 °C; R*_f_* = 0.40 (CH_2_Cl_2_/MeOH, 9:1). IR (KBr): 3335, 3282, 1621, 1344, 1041 cm^−1^. ^1^H-NMR (DMSO-*d*_6_): δ = 2.74 (s, 3H, CH3), 7.85–7.91 (m, 3H, ArH), 8.00–7.95 (m, 1H, ArH), 10.15 ppm (br, 1H, CONH). ^13^C-NMR (DMSO-d6): δ = 110.97, 124.49, 126.44, 128.94, 132.92, 133.85, 134.59, 155.87, 166.44. ESI-HRMS *m/z* = 270.04543 [M+H]^+^. Anal. calcd for C_10_H_11_N_3_O_4_S: C 44.60, H 4.12, N 15.60; found: C 44.62, H 4.11, N 15.44.

General Procedure for Preparation of Compounds **14a**–**h**

A mixture of compound **16** (0.37 mmol) and the corresponding aromatic or heteroaromatic aldehydes (0.37 mmol) in absolute ethanol (10 mL) containing one drop of 37% hydrochloric acid was stirred at room temperature for ca 30 min, until reaction completion (as indicated by TLC). Then the mixture was poured in cold water and filtered. The residue was washed with water and hot hexane and dried under vacuum to produce the desired *N*-acylhydrazone derivatives **14a**–**h**. When necessary, further purification was performed by silica gel column chromatography to give compounds **14a**–**h** with purity of over 98% by HPLC. 

*4-Hydroxy-2-methyl-N’-[(E)-phenylmethylidene]-2H-1,2-benzothiazine-3-carbohydrazide 1,1-dioxide* (**14a**). The title compound was obtained by condensation of **16** with benzaldehyde as a white powder (116 mg, 88%). Mp 223–224 °C; R*_f_* = 0.71 (CH_2_Cl_2_/MeOH, 9:1). IR (KBr): 3281, 1638, 1618, 1341, 1181, 956 cm^−1^. ^1^H-NMR (DMSO-*d*_6_): δ = 2.85 (s, 3H, C*H*_3_), 7.48-7.49 (m, 3H, Ar*H*), 7.74 (d, *J* = 2 Hz, 2H, Ar*H*), 7.90–7.92 (m, 3H, Ar*H*), 8.02-8.05 (m, 1H, Ar*H*), 8.68 (s, 1H, N=C*H*), 11.95 (s, 1H, CON*H*), 14.23 (br, 1H, O*H*). ^13^C-NMR (DMSO-*d*_6_): δ = 111.14, 124.75, 126.79, 127.96, 128.32, 129.48, 131.18, 133.62, 134.10, 134.45, 134.76, 151.47, 158.10, 165.63. ESI-HRMS *m/z* = 358.0856 [M+H]^+^. Anal. calcd. For C_17_H_15_N_3_O_4_S: C 57.13, H 4.23, N 11.76; found: C 57.25, H 4.24, N 11.44. HPLC (C18, acetonitrile-water, 7:3, 254 nm): ret. time: 5.868 min; peak area = 98,299%.

*4-Hydroxy-2-methyl-N'-[(E)-pyridinyl-2-methylidene]-2H-1,2-benzothiazine-3-carbohydrazide 1,1-dioxide* (**14b**). The title compound was obtained by condensation of **16** with 2-pyridinecarboxaldehyde as a light yellow powder (90 mg, 71%). Mp 253–254 °C; R*_f_* = 0.33 (CH_2_Cl_2_/MeOH, 9:1). IR (KBr): 3344, 1666, 1597, 1342, 1177, 962 cm^−1^. ^1^H-NMR (DMSO-*d*_6_): δ = 2.85 (s, 3H, C*H*_3_), 7.44 (m,1H, Ar*H*), 7.85–7.88 (m, 1H, Ar*H*), 7.89–7.90 (t, *J* = 4, 1H, Ar*H*), 7.90–7.94 (m, 1H, Ar*H*), 7.97 (d, *J* = 8, 1H, Ar*H*), 8.02–8.05 (m, 1H, Ar*H*), 8.64 (ddd, *J* = 8 Hz,*J* = 4 Hz, *J* = 2 Hz, 1H, Ar*H*), 8.69 (s, 1H, N=C*H*), 12.14 (s, 1H, CON*H*), 14.09 (br, 1H, O*H*). ^13^C-NMR (DMSO-*d*_6_): δ = 111.13, 120.81, 124.78, 125.35, 126.86, 128.23, 133.75, 134.14, 134.78, 137.53, 150.22, 151.40, 153.44, 158.47, 165.98; ESI-HRMS *m/z* = 359.0809 [M+H]^+^. Anal. calcd for C_16_H_14_N_4_O_4_S: C 53.62, H 3.94, N 15.63; found: C 53.48, H 3.92, N 15.59. HPLC (C18, acetonitrile-water, 45:55, 254 nm): ret. time: 3.916 min; peak area = 98,484%.

*4-Hydroxy-2-methyl-N'-{(E)-[4-(2-propanyl)phenyl]phenylmethylidene}-2H-1,2-benzothiazine-3-carbohydrazide 1,1-dioxide* (**14c**). The title compound was obtained by condensation of **16** with 4-isopropylbenzaldehyde as a white powder (131 mg, 89%). Mp 228–229 °C; R*_f_* = 0.82 (CH_2_Cl_2_/MeOH, 9:1). IR (KBr): 3283, 1638, 1615, 1348, 1182, 958 cm^−1^. ^1^H-NMR (DMSO-*d*_6_): δ = 1.23 (d, *J* = 6 Hz, 6H, CH(C*H*_3_)_2_), 2.84 (s, 3H, C*H*_3_), 2.96 (h, 1H, C*H*(CH_3_)_2_ ), 7.36 (d, *J* = 8 Hz, 2H, Ar*H*), 7.67 (d, *J* = 8 Hz, 2H, Ar*H*), 7.91 (m, 3H, Ar*H*), 8.02–8.06 (m, 1H, Ar*H*), 8.64 (s, 1H, N=C*H*), 11.89 (s, 1H, CON*H*), 14.24 (br, 1H, O*H*). ^13^C-NMR (DMSO-*d*_6_): δ = 24.17, 33.97, 111.14, 124.75, 126.77, 127.47, 128.09, 128.34, 132.14, 133.61, 134.12, 134.74, 151.51, 151.89, 158.01, 165.51. ESI-HRMS *m/z* = 400.1309 [M+H]^+^. Anal. calcd for C_20_H_21_N_3_O_4_S: C 60.13, H 5.30, N 10.52; found: C 60.30, H 5.27, N 10.39. HPLC (C18, acetonitrile-water, 7:3, 254 nm): ret. time: 10.977 min; peak area = 98,127%.

*4-Hydroxy-2-methyl-N'-{(E)-[4-(dimethylamino) phenyl] phenylmethylidene}-2H-1,2-benzothiazine-3-carbohydrazide 1,1-dioxide* (**14d**). The title compound was obtained by condensation of **16** with 4-dimethylaminobenzaldehyde as an orange powder (137 mg, 89%). Mp 217–218 °C; R*_f_* = 0.70 (CH_2_Cl_2_/MeOH, 9:1). IR (KBr): 3287, 1598, 1344, 1181, 956 cm^−1^. ^1^H-NMR (DMSO-*d*_6_): δ = 2.83 (s, 3H, C*H*_3_), 2.99 (s, 6H, N(C*H*_3_)_2_), 6.78 (d, *J* = 8 Hz, 2H, Ar*H*), 7.56 (d, *J* = 8 Hz, 2H, Ar*H*), 7.89-7.93 (m, 3H, Ar*H*), 8.00–8.05 (m, 1H, Ar*H*), 8.52 (s, 1H, N=C*H*), 11.65 (s, 1H, CON*H*), 14.42 (br, 1H, O*H*); ^13^C-NMR (DMSO-*d*_6_): δ = 111.22, 112.43, 121.67, 124.72, 126.65, 128.47, 129.42, 132.14, 133.42, 134.06, 134.68, 152.27, 152.37, 157.56, 164.90. ESI-HRMS *m/z* = 401.1276 [M+H]^+^. Anal. calcd. for C_19_H_20_N_4_O_4_S: C 56.99, H 5.03, N 13.99; found: C 56.69, H 5.03, N 13.99. HPLC (C18, acetonitrile-water, 7:3, 254 nm): ret. time: 4.420 min; peak area = 98,349%.

*4-Hydroxy-2-methyl-N'-[(E)-thiophenyl-2-methylidene]-2H-1,2-benzothiazine-3-carbohydrazide 1,1-dioxide* (**14e**). The title compound was obtained by condensation of **16** with thiophenecarboxaldehyde as a white powder (124 mg, 92%). Mp 236–237 °C; R*_f_* = 0.63 (CH_2_Cl_2_/MeOH, 9:1). IR (KBr): 3267, 1636, 1614, 1338, 1181, 957 cm^−1^.^ 1^H-NMR (DMSO-*d*_6_): δ = 2.83 (s, 3H, C*H*_3_), 7.17 (t,*J* = 4 Hz, 1H, Ar*H*), 7.52 (d, *J* = 2 Hz, 1H, Ar*H*), 7.73 (d, *J* = 4, 1H, Ar*H*), 7.90–7.97 (m, 3H, Ar*H*), 8.01–8.06 (m, 1H, Ar*H*), 8.84 (s, 1H, N=C*H*), 11.91 (s, 1H, CON*H*), 14.16 ppm (br, 1H, O*H*); ^13^C-NMR (DMSO-*d*_6_): δ = 111.12, 124.74, 126.77, 128.31, 128.59, 130.41, 132.53, 133.61, 134.12, 134.70, 139.11, 146.30, 157.98, 165.31; ESI-HRMS *m/z* = 364.0417 [M+H]^+^. Anal. calcd. for C_15_H_13_N_3_O_4_S_2_: C 49.57, H 3.61, N 11.56; found: C 49.58, H 3.61, 11.27. HPLC (C18, acetonitrile-water, 7:3, 254 nm): ret. time: 5.378 min; peak area = 98,866%.

*4-Hydroxy-2-methyl-N'-[(E)-1,3-thiazolyl-2-methylidene]-2H-1,2-benzothiazine-3-carbohydrazide 1,1-dioxide* (**14f**). The title compound was obtained by condensation of **16** with 2-thiazolecarboxaldehyde (0.04 mL, 0.37 mmol) as a white powder (93 mg, 63%). Mp 224–225 °C; R*_f_* = 0.36 (CH_2_Cl_2_/-MeOH, 9:1). IR (KBr) 3223, 1624, 1355, 1182, 948 cm^−1^.^1^H-NMR (DMSO-*d*_6_): δ = 2.85 (s, 3H, C*H*_3_), 7.89 (dd,*J* = 4 Hz, *J* = 2 Hz, 1H, Ar*H*), 7.90–1.95 (m, 3H, Ar*H*), 7.99 (d, *J* = 4, 1H, Ar*H*), 8.03-8.05 (m, 1H, Ar*H*), 8.85 (s, 1H, N=C*H*), 12.23 (s, 1H, CON*H*), 13.93 (br, 1H, O*H*). ^13^C-NMR (DMSO-*d*_6_): δ = 110.55, 122.63, 124.25, 125.35, 127.58, 133.28, 133.62, 134.18, 144.28, 144.70, 158.01, 163.76, 165.23. ESI-HRMS *m/z =* 365.0378 [M+H]^+^. Anal. calcd. for C_14_H_12_N_4_O_4_S_2_: C 46.14, H 3.32, N 15.38; found C 46.03, H 3.39, N 15.42. HPLC (C18, acetonitrile-water, 45:55, 254 nm): ret. time: 11.831 min; peak area = 99,751%.

*4-Hydroxy-2-methyl-N'-[(E)-4-biphenylphenylmethylidene]-2H-1,2-benzothiazine-3-carbohydrazide 1,1-dioxide* (**14g**). The title compound was obtained by condensation of **16** with 4-biphenylcarboxaldehyde as a light green powder (146 mg, 91%). Mp 215–216 °C; R*_f_* = 0.72 (CH_2_Cl_2_/MeOH, 9:1). IR (KBr): 3271, 1613, 1343, 1182, 959 cm^−1^. ^1^H-NMR (DMSO-*d*_6_): δ = 2.86 (s, 3H, C*H*_3_), 7.36–7.53 (m, 3H, Ar*H*), 7.74 (d, *J* = 8 Hz, 2H, Ar*H*), 7.81–7.82 (m, 3H, Ar*H*), 7.87–7.93 (m, 4H, Ar*H*), 8.03–8.06 (m, 1H, Ar*H*), 8.72 (s, 1H, N=C*H*), 12.01 (s, 1H, CON*H*), 14.24 (br, 1H, O*H*). ^13^C-NMR (DMSO-*d*_6_): δ = 111.15, 124.76, 126.80, 127.28, 128.33, 128.59, 129.60, 133.54, 133.65, 134.13, 134.74, 139.79, 142.66, 150.99, 158.13, 165.62.; ESI-HRMS *m/z* = 434.1165 [M+H]^+^. Anal. calcd. for C_23_H_19_N_3_O_4_S: C 63.73, H 4.42, N 9.69; found: C 63.87, H 4.39, N 9.65. HPLC (C18, acetonitrile-water, 9:1, 254 nm): ret. time: 4.653 min; peak area = 99,145%.

*4-Hydroxy-2-methyl-N'-{(E)-[3,5-di-terc-buthyl-4-hydroxy]phenylmethylidene}-2H-1,2-benzothiazine-3-carbohydrazide 1,1-dioxide* (**14h**). The title compound was obtained by condensation of **16** with 3,5-di-tert-buthyl-4-hydroxyphenylcarboxaldehyde as a light green powder (161 mg, 90%). Mp 249–251 °C; R*_f_* = 0.61 (CH_2_Cl_2_/MeOH, 9:1). IR (KBr): 3287, 1645, 1617, 1348, 1183, 954 cm^−1^. ^1^H-NMR (DMSO-*d*_6_): δ = 1.42 (s, 18H, C(C*H*_3_)_3_), 2.83 (s, 3H, C*H*_3_), 7.49 (s, 2H, Ar*H*), 7.53 (s, 1H, O*H*), 7.91–8.02 (m, 4H, Ar*H*), 8.59 (s, 1H, N=C*H*), 11.71 (s, 1H, CON*H*), 14.25 (br, 1H, O*H*); ^13^C-NMR (DMSO-*d*_6_): δ = 30.11, 34.50, 110.64, 124.30, 125.11, 126.16, 134.16, 139.24, 152.42, 156.66, 157.09, 164.51. ESI-HRMS *m/z* = 486.2056 [M+H]^+^. Anal. calcd for C_25_H_31_N_3_O_5_S: C 61.83, H 6.43, N 8.65; found: C 61.93, H 6.39, N 8.61. HPLC (C18, acetonitrile-water, 9:1, 254 nm): ret. time: 5.333 min; peak area = 99,697%.

### 3.2. X-ray Crystallography

A colorless prismatic single crystal of compound **14a** (LASSBio-1606) suitable for x-ray study was obtained by slow evaporation of a solution of dichloromethane-dimethyl sulfoxide (1:15) at room temperature 295(2) K. Data collection was performed using the Enraf-Nonius CAD-4 diffractometer operating with Cu-Kα radiation at room temperature. 3718 data points were collected of what 3004 are symmetry independent (R_int_ = 0.0267). The molecule crystallizes in the C2/c space group, having Z = 8. Structure solution was obtained using Direct Methods implemented in SHELXS [52] and the model refinement was performed with full matrix least squares on F^2^ using SHELXL [52], with final residuals R1 = 0.056, wR2 = 0.148 for 2633 observed data with I > 2σ(I), and R1 = 0.071, wR2 = 0.299 for all data. The crystal packing is mediated by a pair of intermolecular hydrogen short contact of type N17−H17…O12^i^ with donor-acceptor distance 3.156(3) Å and DHA angle 149.3°, forming dimmers about an inversion center, and further stabilization is due to weak interactions of types C10—H…O12^ii^, C13—H…O15^iii^ and C22—H…O11^iv^. Hydrogen interactions geometry is given in Table 6. The programs ORTEP-3 [39], SHELXS/SHELXL [52] were used within WinGX [53] software package.

**Table 6:** Intramolecular and intermolecular hydrogen bonds and weak interactions.

D—H...A	D—H (Å)	H...A (Å)	D...A (Å)	D—H...A (°)	Symmetry operation
N17-H17...N2	0.86	2.33	2.723(3)	108.1	
O16-H16...O15	0.82	1.81	2.541(3)	147.4	
N17-H17...O12 ^i^	0.86	2.39	3.156(3)	149.3	(i) 1/2-x, 1/2-y, -z
C10-H10...O12 ^ii^	0.93	2.69	3.268(4)	121.3	(ii) 1-x, -y, -z
C13-H13B...O15 ^iii^	0.96	2.55	3.466(4)	158.7	(iii) 1-x, 1-y, -z
C22-H22...O11 ^iv^	0.93	2.48	3.373(4)	162.3	(iv) x, 1-y, -1/2+z

### 3.3. Pharmacological Evaluation

#### 3.3.1. Animals

Swiss mice weighing 20–30 g (from the BIOCEN-UFAL) were housed in group cages and maintained on a 12 h light/12 h dark cycle. Animals had free access to food and water at all times. Experiments were carried out according to a protocol approved by the Animal Welfare Committee of Federal University of Alagoas (UFAL) (Number: 026681/2009-23), and in according with the ethical guidelines for investigation of experimental pain in conscious animals

#### 3.3.2. Reagents

Acetic acid (Merck), arabic gum (Sigma Aldrich), morphine sulphate (Dimorf-Cristalia-BR) and piroxicam (Merck) were obtained from commercial sources. A solution of formalin 2.5% was prepared with formaldehyde (Merck) in saline (NaCl 0.9%). Piroxicam and compounds **14a**–**h** were used as suspension in arabic gum in all the experiments and oral administrations.

#### 3.3.3. Acetic Acid-induced Writhing Test

This test was performed as described by Collier *et al.* [40]. Acetic acid (0.6%, v/v) was administered i.p. in a volume of 0.1 mL/10 g. The number of writhes, a response consisting of contraction of an abdominal wall, pelvic rotation followed by hind limb extension, was counted during continuous observation for 20 min beginning from 5 min after the acetic acid injection. Piroxicam and compounds **14a**–**h** (all 100 µmol/kg, oral administration) were administered 60 min before the acetic acid injection. Antinociceptive activity was expressed as inhibition percent of the usual number of writhing observed in control animals. Dose–response curves were obtained for piroxicam and LASSBio-1604 (**14e**) (1, 10, 30, 100, 300 µmol/kg), LASSBio-1617 (**14b**) (1, 10, 30, 100, 300 µmol/kg), LASSBio-1637 (**14f**) (1, 10, 30, 100, 300 µmol/kg), LASSBio-1638 (**14g**) (1, 10, 30, 100, 300 µmol/kg), and LASSBio-1639 (**14h**) (1, 10, 30, 100, 300 µmol/kg) using groups of 8 animals. Control animals received the vehicle. The ID_50_ values (*i.e.*, dose which reduces response by 50% relative to the control values) of piroxicam, of compounds **14e**, **14a**, **14f**, **14g** and **14h** were determined by linear regression from individual experiments with the linear regression function of the Graph Pad Prisma software.

#### 3.3.4. Formalin-induced Nociception

The procedure used was essentially the same as that described previously [41]. Animals received 20 mL of 2.5% formalin solution (0.92% formaldehyde in saline) in the ventral surface of the right hind paw. Animals were observed from 0 to 5 min (neurogenic phase) and from 15 to 30 min (inflammatory phase) and the time that they spent licking the injected paw was recorded and considered as indicative of nociception. Animals received piroxicam or compounds **14a**–**h** (100 μmol/kg, oral administration) 40 min beforehand. Control animals received vehicle (arabic gum).

#### 3.3.5. Hot-plate Test

Mice were treated according to the method described by Kuraishi *et al.* [46]. Animals (n = 6) were placed on a hot-plate set at 55 ± 1 °C. Reaction time was recorded when the animals licked their fore and hind-paws and jumped at 30, 60, 90 and 120 min after oral administration of 100 µmol/kg of piroxicam or compounds **14a**–**h** or reference drug (morphine, 15 µmol/kg. i.p.). Baseline was considered as the mean of reaction time obtained at 30 and 60 min before administration of derivatives or piroxicam or morphine and was defined as normal reaction of animal to the temperature.

#### 3.3.6. Zymosan-induced Peritonitis

Peritoneal inflammation was induced according to the method described by Leite *et al.* [49]. A solution of Zymosan A (Sigma–Aldrich) (2 mg/mL) was prepared in saline (NaCl 0.9%) and injected into the peritoneal cavity of mice (0.5 mL). Six hours after injection of Zymosan A, the animals were killed by cervical dislocation and the peritoneal cavity was washed with 3 mL of cold Hank’s. Compounds **14a**–**h** or piroxicam were administered at the dose of 100 µmol/kg (p.o.), 40 min before Zymosan A injection. Control group received 10 mL/kg of vehicle (arabic gum, p.o.). The number of cells was quantified by optical microscope, using the 100° lens.

#### 3.3.7. Carrageenan-induced Peritonitis

Peritoneal inflammation was induced according to the method described by Ferrandiz and Alcaraz [50]. A solution of carrageenan 1% (Sigma-Aldrich) was prepared in saline (NaCl 0.9%) and injected into the peritoneal cavity of mice (250 µL/animal). Four hours after injection of carrageenan, the animals were killed by cervical dislocation and the peritoneal cavity was washed with 3 mL of cold Hank’s. Compounds **14a**–**h** and piroxicam were administered at the dose of 100 µmol/kg (p.o.), 30 min before carrageenan injection. Control group received 10 mL/kg of vehicle (arabic gum, p.o.). The number of cells was quantified by optical microscope, using 100× lens.

#### 3.3.8. Evaluation of Human COX-1/COX-2 Inhibition

Evaluation of piroxicam and compounds **14b**,**e** for COX-1 and COX-2 inhibition were performed by CEREP Laboratories (Celle L’Evescault, France) using, respectively, assay catalog reference no. 0726 and 0727, and both were carried out as described by Glaser *et al.* [51]. In these assays, the inhibitory effect of compounds, at concentration of 10 μM, on activity of human recombinant COX-1 or COX-2, isolated from Sf-9 cells, was quantified by measuring the formation of PGE_2_, detected by homogeneous time resolved fluorescence (HTRF), from arachidonic acid. The results are expressed as a percent inhibition of the control enzyme activity.

#### 3.3.9. Test Evaluation of the Inhibition of LOX

Compounds **14b**, **14e**–**h** and piroxicam were evaluated for their ability to inhibit LOX, using the kit for determining the inhibition of LOX (Lipoxygenase Inhibitor Screening Assay Kit, Cayman Chemical Company, Ann Arbor, MI, USA) according to manufacturer's instructions. The test compound solutions were prepared using DMSO as solvent and reaction buffer (supplied in kit) to a concentration of 10 µM.

#### 3.3.10. Statistical Analysis

Data obtained from animal experiments are represented by mean ± standard error of the mean (Mean ± S.E.M.). Statistical differences between the treated and the control groups were evaluated by test t of Student or ANOVA in the tutorial Prisma®. Values were considered significant if * *p* < 0.05 and ** *p* < 0.01. The ID_50_ values (*i.e.*, the dose of derivatives *N*-acylhydrazone which reduced the pain response by 50% in relation to control group values) were determined by linear regression from individual experiments using the GraphPad software (GraphPad Software, San Diego, CA, USA) and are reported as geometric means accompanied by their respective 95% confidence limits. Maximal inhibition values were calculated at the more effective dose used.

## 4. Conclusions

In general, compounds **14a**–**h** presented antinociceptive and anti-inflammatory activities *in vivo*, by oral administration, at the screening dose of 100 µmol/kg. Pharmacological evaluation suggest that the NAH derivatives reported herein present a better pharmacological profile than standard drug piroxicam, especially for their markedly activity in acute inflammation models. Moreover, we were able to identify LASSBio-1637 (**14f**) and LASSBio-1639 (**14g**) as new antinociceptive and anti-inflammatory prototypes, which are able to inhibit cell recruitment in carrageenan and zymosan-induced peritonitis in more than 70% and 80% (dose = 100 μmol/kg, p.o), respectively, through a mechanism of action that seems to be distinct of piroxicam and remains to be elucidated.

## Supplementary Material

The structural results for **14a** were deposited with the Cambridge Crystallographic Data Centre as Supporting Information, CCDC number: 908020. Copies of the data can be obtained free of charge upon application to CCDC, 12 Union Road, Cambridge CB2 1EZ, United Kingdom (Fax: (44) 1223 336-033; E-mail: deposit@ccdc.cam.ac.uk).

Supplementary materials can be accessed at: http://www.mdpi.com/1420-3049/17/12/14126/s1.
