# Supplementary material for: Design, Synthesis, Antinociceptive and Anti-Inflammatory Activities of Novel Piroxicam Analogues
Source: Molecules. 2012 Nov 28;17(12):14126–45. doi: 10.3390/molecules171214126 (PMC6268254; doi:10.3390/molecules171214126)

checkCIF/PLATON report

Structure factors have been supplied for datablock(s) I

No syntax errors found. CIF dictionary Interpreting this report

Datablock: I

```
Bond precision:  C-C = 0.0043 A          Wavelength=1.54184

Cell:            a=15.693(1)    b=11.628(2)        c=19.789(3)
                 alpha=90       beta=113.624(7)    gamma=90
Temperature:     295 K

                Calculated                   Reported
Volume          3308.4(8)                    3308.4(8)
Space group     C 2/c                        C 2/c
Hall group      -C 2yc                       -C 2yc
Moiety formula  C17 H15 N3 O4 S              C17 H15 N3 O4 S
Sum formula     C17 H15 N3 O4 S              C17 H15 N3 O4 S
Mr              357.39                       357.39
Dx,g cm-3       1.435                        1.435
Z               8                            8
Mu (mm-1)       1.993                        1.993
F000            1488.0                       1488.0
F000'           1495.20
h,k,lmax        18,13,23                     18,13,23
Nref            3004                         3004
Tmin,Tmax       0.733,0.742                  0.572,0.737
Tmin'           0.388

Correction method= PSI-SCAN

Data completeness= 1.000          Theta(max)= 67.840

R(reflections)= 0.0560( 2633)     wR2(reflections)= 0.1986( 3004)

S = 1.226                   Npar= 227
```

The following ALERTS were generated. Each ALERT has the format
test-name_ALERT_alert-type_alert-level.
Click on the hyperlinks for more details of the test.

Alert level B

```
PLAT934_ALERT_3_B Number of (Iobs-Icalc)/SigmaW .gt. 10 Outliers .          9
PLAT939_ALERT_3_B Large Value of Not (SHELXL) Weight Optimized S .     282.50
```

Alert level C

```
PLAT340_ALERT_3_C Low Bond Precision on  C-C Bonds ...............     0.0043 Ang
PLAT918_ALERT_3_C Reflection(s) # with I(obs) much smaller I(calc)          7
PLAT975_ALERT_2_C Positive Residual Density at 1.06A from N17    .       0.49 eA-3
```

Alert level G

```
PLAT005_ALERT_5_G No _iucr_refine_instructions_details  in CIF ...          ?
PLAT007_ALERT_5_G Note: Number of Unrefined D-H Atoms ............          2
PLAT072_ALERT_2_G SHELXL First  Parameter in WGHT Unusually Large.       0.13
PLAT909_ALERT_3_G Percentage of Observed Data at Theta(Max) still          80 Perc.
```

---

0 **ALERT level A** = Most likely a serious problem - resolve or explain
2 **ALERT level B** = A potentially serious problem, consider carefully
3 **ALERT level C** = Check. Ensure it is not caused by an omission or oversight
4 **ALERT level G** = General information/check it is not something unexpected

0 ALERT type 1 CIF construction/syntax error, inconsistent or missing data
2 ALERT type 2 Indicator that the structure model may be wrong or deficient
5 ALERT type 3 Indicator that the structure quality may be low
0 ALERT type 4 Improvement, methodology, query or suggestion
2 ALERT type 5 Informative message, check

---

checkCIF publication errors

Alert level A

```
PUBL006_ALERT_1_A  _publ_requested_journal is missing
            e.g. 'Acta Crystallographica Section C'
PUBL012_ALERT_1_A  _publ_section_abstract is missing.
            Abstract of paper in English.
PUBL024_ALERT_1_A The number of authors is greater than 5.
              Please specify the role of each of the co-authors
              for your paper.
```

Alert level G

```
PUBL013_ALERT_1_G The _publ_section_comment (discussion of study) is
            missing. This is required for a full paper submission (but is
            optional for an electronic paper).
PUBL017_ALERT_1_G The _publ_section_references section is missing or
            empty.
```

---

3 **ALERT level A** = Data missing that is essential or data in wrong format
2 **ALERT level G** = General alerts. Data that may be required is missing

---

Publication of your CIF

You should attempt to resolve as many as possible of the alerts in all categories. Often the minor alerts point to easily fixed oversights, errors and omissions in your CIF or refinement strategy, so attention to these fine details can be worthwhile. In order to resolve some of the more serious problems it may be necessary to carry out additional measurements or structure refinements. However, the nature of your study may justify the reported deviations from journal submission requirements and the more serious of these should be commented upon in the discussion or experimental section of a paper or in the "special_details" fields of the CIF. *checkCIF* was carefully designed to identify outliers and unusual parameters, but every test has its limitations and alerts that are not important in a particular case may appear. Conversely, the absence of alerts does not guarantee there are no aspects of the results needing attention. It is up to the individual to critically assess their own results and, if necessary, seek expert advice.

If level A alerts remain, which you believe to be justified deviations, and you intend to submit this CIF for publication in Acta Crystallographica Section C or Section E, you should additionally insert an explanation in your CIF using the Validation Reply Form (VRF) below. Your explanation will be considered as part of the review process.

If you intend to submit to another section of Acta Crystallographica or Journal of Applied Crystallography or Journal of Synchrotron Radiation, you should make sure that at least a basic structural check is run on the final version of your CIF prior to submission.

```
# start Validation Reply Form
_vrf_PUBL006_GLOBAL
;
PROBLEM: _publ_requested_journal is missing
RESPONSE: ...
;
_vrf_PUBL012_GLOBAL
;
PROBLEM: _publ_section_abstract is missing.
RESPONSE: ...
;
_vrf_PUBL024_GLOBAL
;
PROBLEM: The number of authors is greater than 5.
RESPONSE: ...
;
# end Validation Reply Form
```

If you wish to submit your CIF for publication in Acta Crystallographica Section C or E, you should upload your CIF via the web. If your CIF is to form part of a submission to another IUCr journal, you will be asked, either during electronic submission or by the Co-editor handling your paper, to upload your CIF via our web site.

PLATON version of 22/10/2012; check.def file version of 16/10/2012

Datablock I - ellipsoid plot

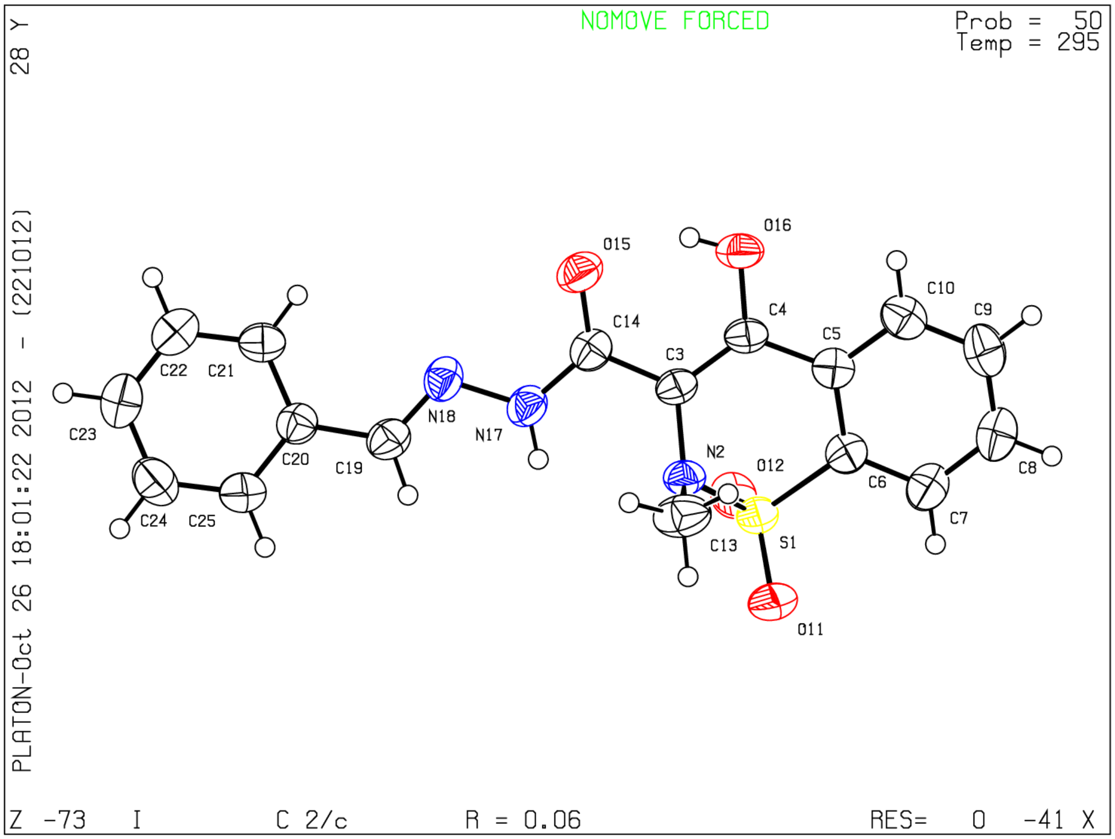

Supplement: Supplementary file 1 [file molecules-17-14126-s001.pdf]
